# Common mental disorders and care provided by the Health Care Network for the rural population: a cross-sectional study, Ceará, Brazil, 2020

**DOI:** 10.1590/S2237-96222026v35e20250540.en

**Published:** 2026-07-20

**Authors:** Luis Lopes Sombra, Eugênio de Moura Campos, Ricardo José Soares Pontes, Vanira Matos Pessoa

**Affiliations:** 1 Universidade Federal do Ceará Fortaleza Fortaleza CE Brazil Universidade Federal do Ceará, Fortaleza, CE, Brazil; 2 Fundação Oswaldo Cruz Ceará Eusébio Eusébio CE Brazil Fundação Oswaldo Cruz Ceará, Eusébio, CE, Brazil

**Keywords:** Mental Health, Rural Health, Primary Health Care, Health Service Needs and Demands, Cross-Sectional Studies, Salud Mental, Salud Rural, Atención Primaria de Salud, Necesidades y Demandas de Servicios de Salud, Estudios Transversales

## Abstract

**Objective:**

To analyze association between presence of common mental disorders (CMD) and care provided by the Health Care Network to service users in rural areas of Ceará state, Brazil.

**Methods:**

This was a cross-sectional study with a convenience sample from the municipalities of Nova Oriente and Icapuí, Ceará, Brazil. After a pilot study, we administered a Health Care Network assessment form and a Self-Reporting Questionnaire for screening for CMD, with a cutoff score of ≥7. Sample size calculation and statistical analysis were performed, including the chi-square test, prevalence ratios (PR), confidence intervals (95% CI), and p-values.

**Results:**

277 service users participated in the study. The mental health disorder prevalence rate was 21.3%. Of these, 17% had a previous diagnosis, 13.5% were under exclusive care in a public service, and 3.3% received all psychotropic medications free of charge. Presence of CMD was associated with consulting with healthcare professionals in the last 12 months (PR 2.46; 95%CI 1.37; 4.42; p-value 0.001), previous diagnosis(es) of health problem(s) made by a professional (PR 1.87; 95%CI 1.13; 3.09; p-value 0.011), follow-up type exclusively private/complementary (PR 1.84; 95%CI 1.08; 3.11; p-value 0.004), and overall assessment of the municipality’s public health service as poor/very poor (PR 2.19; 95%CI 1.24; 3.87; p-value 0.025). 71.5% of participants cited health centers as their most frequently used service, and 40% reported lack of health professional(s).

**Conclusion:**

There is evidence of association between CMD in the rural population and gaps in diagnosis, follow-up and treatment offered, which reinforces the need to expand the mental health care capacity of the Health Care Network, especially in Primary Care.

## Resumen

**Objetivo:**

Analizar la asociación entre la presencia de trastornos mentales comunes (TMC) y la atención ofrecida por la Red de Atención a la Salud a usuarios en zonas rurales del estado de Ceará, Brasil.

**Métodos:**

Estudio transversal con una muestra por conveniencia de los municipios de Nova Oriente e Icapuí, Ceará, Brasil. Tras un estudio piloto, se aplicó un formulario de evaluación de la Red de Atención a la Salud y un Cuestionario de Autoinforme para la detección de TMC, con un puntaje de corte ≥7. Se realizó el cálculo del tamaño de la muestra y el análisis estadístico, incluyendo la prueba de chi-cuadrado, la razón de prevalencia (RP), el intervalo de confianza (IC del 95%) y la generación del valor p.

**Resultados:**

Participaron en el estudio 277 usuarios, con una prevalencia del 21,3% de trastornos de salud mental. De ellos, el 17% contaba con un diagnóstico previo, el 13,5% recibía atención exclusiva en un servicio público y el 3,3% recibía todos los psicofármacos de forma gratuita. La presencia de TMC se asoció con la búsqueda por profesionales de la salud en los últimos 12 meses (RP 2,46; IC95% 1,37; 4,42; valor p 0,001), diagnóstico(s) previo(s) de problema(s) de salud realizado(s) por un profesional (RP 1,87; IC95% 1,13; 3,09; valor p 0,011), ubicación del seguimiento exclusivamente privado/complementario (RP 1,84; IC95% 1,08; 3,11; valor p 0,004) y evaluación general del servicio de salud pública del municipio como malo/muy malo (RP 2,19; IC95% 1,24; 3,87; valor p 0,025). El 71,5% de los participantes citó el centro de salud como el servicio utilizado con mayor frecuencia y el 40% informó falta de profesional(es).

**Conclusión:**

Existe evidencia de una asociación entre TMC en la población rural y las deficiencias en el diagnóstico, seguimiento y tratamiento ofrecidos, lo que refuerza la necesidad de ampliar la capacidad de la Red de Atención a la Salud para la atención de la salud mental, especialmente en Atención Primaria.

**Ethical aspects**

This research respected ethical principles, having obtained the following approval data:

Research ethics committee Escola de Saúde Pública do Ceará

Opinion number 3,372,478

Approval date 5/6/19

Certificate of submission for ethical appraisal 07802419.2.0000.5037

Informed consent form Obtained from all the research participants.

## Introduction

In the 1970s and 1980s, driven by the human rights efforts of health service users, families and health professionals, mainly through the Anti-Asylum Movement, the mental health care model in Brazil underwent profound changes, resulting in the Psychiatric Reform. These changes, in addition to overcoming longstanding prejudices and stigmas related to patients with mental disorders, were fundamental for the adequate management of mental health problems among Brazilians. The Brazilian psychiatric reform brought a new reality to mental health care and, among other advances, made it possible for service users with mental disorders to be cared for within society (1).

The 1988 Constitution of the Federative Republic of Brazil included a significant number of social rights to meet the fundamental needs of the Brazilian population. The right to health emerged as an effect of the very principle of the dignity of the human person, which embodies one of the foundations of the democratic rule of law, determining that each and every citizen has the right to access health services, regardless of gender, class, race/skin color, age or other characteristics. It became the State’s duty to promote public policies in order to enable universal and equal access to the actions necessary for the promotion, protection and recovery of health, culminating in Law No. 8080, which consolidated the creation of the Brazilian Unified Health System (*Sistema Único de Saúde*, SUS) (2,3).

This reality brought about an innovative process of mental health management and care and transformed a system basically focused on hospitalizations in psychiatric hospitals (asylums) into a new approach, based on the quest for the reintegration of patients into the family dynamic, with follow-up in the Health Care Network, including the Psychosocial Care Network, composed of integrated care in various facilities, such as psychosocial care centers, emergency/urgent care services, beds in hospitals and clinics, therapeutic residences and primary health care centers (1,4,5).

In this care model, the organizational basis and primary entry point into the SUS is Primary Health Care (PHC), which includes the Family Health Strategy, responsible for including the family as the object and subject of care linked to the longitudinal care provided by health professionals. Therefore, PHC must consider the specificities of each territory and be integrated into the Health Care Network in order to adequately perform the functions of resolving and coordinating physical and mental health care (6).

One of the major challenges to be faced today is mental illness, particularly the so-called common mental disorders (CMDs). CMDs are composed of symptoms such as insomnia, forgetfulness, difficulty concentrating, discouragement, fatigue and feelings of worthlessness, which cause psychological suffering and disrupt the individual’s normal functioning, despite not meeting the criteria for traditional mental diagnoses. These disorders significantly compromise the lives of affected individuals and directly interfere in multiple dimensions, such as the ability to work, maintain social relationships, take care of oneself and make decisions. It has been projected that, by 2030, mental disorders will be the most disabling health problem for human beings (7). CMD prevalence in populations of different countries, although still underdiagnosed, is between 29% and 35% (8).

Despite the proven importance of primary health care for mental health promotion, prevention and treatment, as well as rehabilitation of mental disorders, weaknesses are still observed, such as: poor integration between services, insufficient multidisciplinary work, weakness in comprehensive care and difficulty in case resolution (9). These limitations interfere with mental health care for the Brazilian population, especially the rural population, which has poorer indicators of access to health services (8). When comparing follow-up of patients diagnosed with depression in both rural and urban territories in 2019, it was observed that, in rural areas, 48% of people received medical assistance for this condition in the last 12 months, while 53% received it in urban territories (10).

The way health services are organized also constitutes a social determinant of health and can contribute to living and health conditions, especially in populations with greater vulnerabilities, such as those who live and survive in rural areas. Rural health cannot be separated from the functioning of public and collective health, especially in developing countries like Brazil, which need to make rational and efficient use of available resources (11). As an aggravating factor for these situations of vulnerability, problems related to mental health are still not fully addressed or solved by the current national health systems (12). The relevance of this study is shown in its production of health indicators on a historically stigmatized and vulnerable population segment.

The objective of this study was to analyze association between the presence of CMDs and care offered by the Health Care Network to service users in rural municipalities of Ceará state, Brazil.

## Methods

### Study design and setting

This is a cross-sectional study with a quantitative approach conducted with residents of the municipalities of Novo Oriente and Icapuí, located in Ceará state, Brazil, and classified by the Brazilian Institute of Geography and Statistics (13) as adjacent rural areas. The criteria used were: population density, access to large urban centers, structuring of goods/services and location of the municipality. In 2020, Novo Oriente had a population of 27,453 inhabitants, while that of Icapuí was 18,392, with estimated rural population percentages of 48.1% and 68.5%, respectively. Table 1 shows the components of the Health Care Network of the municipalities (13,14,15).

**Table 1:** Components (n) of the Health Care Network. Ceará, 2020

Components		Novo Oriente n	Icapuí n
Primary Care	Primary Health Care Centers (rural centers)	13 (08)	08 (05)
	Family Health Strategy Teams	11	08
	Community Health Agents	71	37
	Family Health Support Centers	01	02
Secondary Care	Specialty Centers	02	00
	Urgent Health Care Centers	01	01
	Psychosocial Care Centers	01	01
Teriary Care	General Hospitals	01	01

### Participantes

Convenience sampling with the following inclusion criteria: individuals aged between 18 and 64 years; economically active with productive activity – family farming, artisanal fishing or both activities; resident in the hinterland/countryside and coastal/water areas for more than two years; members of a family assigned to a rural family health team.

Rural workers who were not undertaking their work activities were excluded from the study. Due to the COVID-19 pandemic in 2020, a biosafety protocol was created for data collection in the field, in order to reduce the risks of viral transmission, so that the following exclusion criteria were added: individuals with flu-like symptoms; individuals with body temperature ≥37.8°C.

### Variables

The outcome variable “presence of CMD” was screened using the Self-Reporting Questionnaire, an instrument created by the World Health Organization and derived from international research. This questionnaire has been translated, tested and validated in Brazil and aims to screen individuals with CMDs. It has 62.9% to 90% sensitivity and 44% to 95% specificity. It is a questionnaire composed of 20 questions with “yes” or “no” alternatives, among which four are about physical symptoms and 16 about psycho-emotional symptoms. The standardized cutoff point for positive screening in most studies is seven affirmative answers (16).

The sociodemographic data used were: sex; age group; marital status; race/skin color; religion; work; monthly family income. In order to develop the care cascade, the independent variables of previous diagnosis(es), type of health follow-up service, and medication(s) in continuous use were analyzed. From these general data, specifications related to mental health were made: diagnosis of mental disorder(s); type of mental health follow-up service; psychotropic medication(s) in continuous use. The independent variables regarding the health care offered in the health care network were analyzed based on the following questions: “Have you consulted a health professional in the last 12 months?”; “Have you received a diagnosis(es) of health problem(s) made by a health professional?”; “Where do you receive health follow-up?”; “How do you obtain the medications you take daily?”; “Overall, how do you rate the public health service in your municipality?”; “How do you rate your health status?”.

### Data sources and measurement

The sample was composed by means of identification and mobilization of key informants from the community, members of social movements, taking into account the study inclusion and exclusion criteria. Data collection took place during September and October 2020 using a form consisting of questions about sociodemographic data and assessment of health services, as well as administration of the Self-Reporting Questionnaire instrument.

The questionnaire was developed by the researchers after an eight-hour workshop with representatives of social movements from rural and water-based communities. Subsequently, the questionnaire underwent initial standardization and was administered by two researchers to four individuals, two men and two women. Based on the demands experienced by the researchers during this initial administration, the questionnaire underwent revision processes.

At the time of the interview, mobile devices containing the form were used via the Open Data Kit application. The data input to the application automatically generated a virtual table on a storage platform, which was monitored virtually by a researcher throughout the data collection period.

### Bias

The study had biases inherent to its cross-sectional design, as well as limitations related to its implementation in only two municipalities of a single Brazilian state, which may restrict the generalizability of the results. Using convenience sampling also reduces the statistical representativeness of the findings. Nevertheless, this research offers important contributions by deepening the understanding of healthcare in rural contexts, allowing for identification of local patterns and production of contextualized evidence that can support public policies and actions more sensitive to the realities of vulnerable territories.

### Study size 

The sample size calculation was performed using the following parameters: 95% confidence level, 5% sampling error, and an estimated proportion of the outcome variable in the population of 22.58%, based on a pilot test conducted in a rural municipality in the same state with sociodemographic indicators similar to the municipalities selected for this study (13,14,15). That pilot study took place in Fortim, Ceará state, with a sample of 31 individuals living in rural areas, whereby CMD prevalence was found to be 22.58% (n=7). The estimated minimum sample size for our study was 269 individuals.

### Statistical methods

The table generated with the data collected was input to the Statistical Package for the Social Sciences (SPSS) program for descriptive statistical analysis, with estimation of absolute (n) and relative (%) frequencies, and analytical statistical analysis.

The statistical analysis was performed using Pearson’s chi-square test (X2) or Fisher’s exact test to identify association between each of the independent variables related to assessment of health care and the dichotomous dependent variable of individuals with CMDs. Prevalence ratios (PR) and 95% confidence intervals (95%CI) were also used, thus generating their p-values, whereby a p-value<0.05 was taken to be statistically significant.

## Results

The total sample of 277 participants had the following sociodemographic profile: predominantly female (69.7%), age range between 51-64 years (36.1%), average age 43 years, married/stable union (79.8%). Regarding self-reported race/skin color, 67.5% identified as mixed-race, 19.5% as White, and 10.1% as Black. 64.2% identified as Catholic, 60.7% had artisanal fishing as their main occupation, and 64.3% had a monthly family income of up to one minimum wage (Table 2).

**Table 2:** Absolute (n) and relative (%) sociodemographic distribution of Health Network users. Ceará, 2020 (n=277)

Variables	n (%)
Sex	
Male	84 (30.3)
Female	193 (69.7)
**Age group **(years)	
18-30	47 (17)
31-40	55 (19.8)
41-50	75 (27.1)
51-64	100 (36.1)
**Marital status**	
Married/Stable union	221 (79.8)
Single	39 (14.1)
Divorced	13 (4.7)
Widowed	4 (1.4)
**Race/skin color**	
Mixed race	187 (67.5)
White	54 (19.5)
Black	28 (10.1)
Asian	7 (2.5)
Indigenous	1 (0.4)
Religion	
Catholic	178 (64.2)
Evangelical	50 (18.1)
None	49 (17.7)
Work	
Family farming	32 (11.5)
Artisanal fishing	168 (60.7)
Family farming and Artisanal fishing	77 (27.8)
**Monthly family income **(**minimum wages**)	
Up to 1	151 (64.3)
1-2	39 (16.6)
More than 2	45 (19.1)

A 21.3% prevalence rate was found for service users with CMDs. Only 3.6% reported having been diagnosed as having a mental disorder, namely anxiety and/or depression. All of these service users reported being in follow-up and 80% (n=8) indicated that they were exclusively receiving care in public services. All were on continuous treatment with psychotropic drugs and 75% (n=6) needed to purchase some of their medication.

The care cascade for service users who tested positive for CMDs showed that only 17% had previously been diagnosed by a healthcare professional as having a mental disorder, 13.5% (n=8) reported exclusive follow-up in a public health service, and 3.3% received all psychotropic medications free of charge (Figure 1).

**Figure 1:**
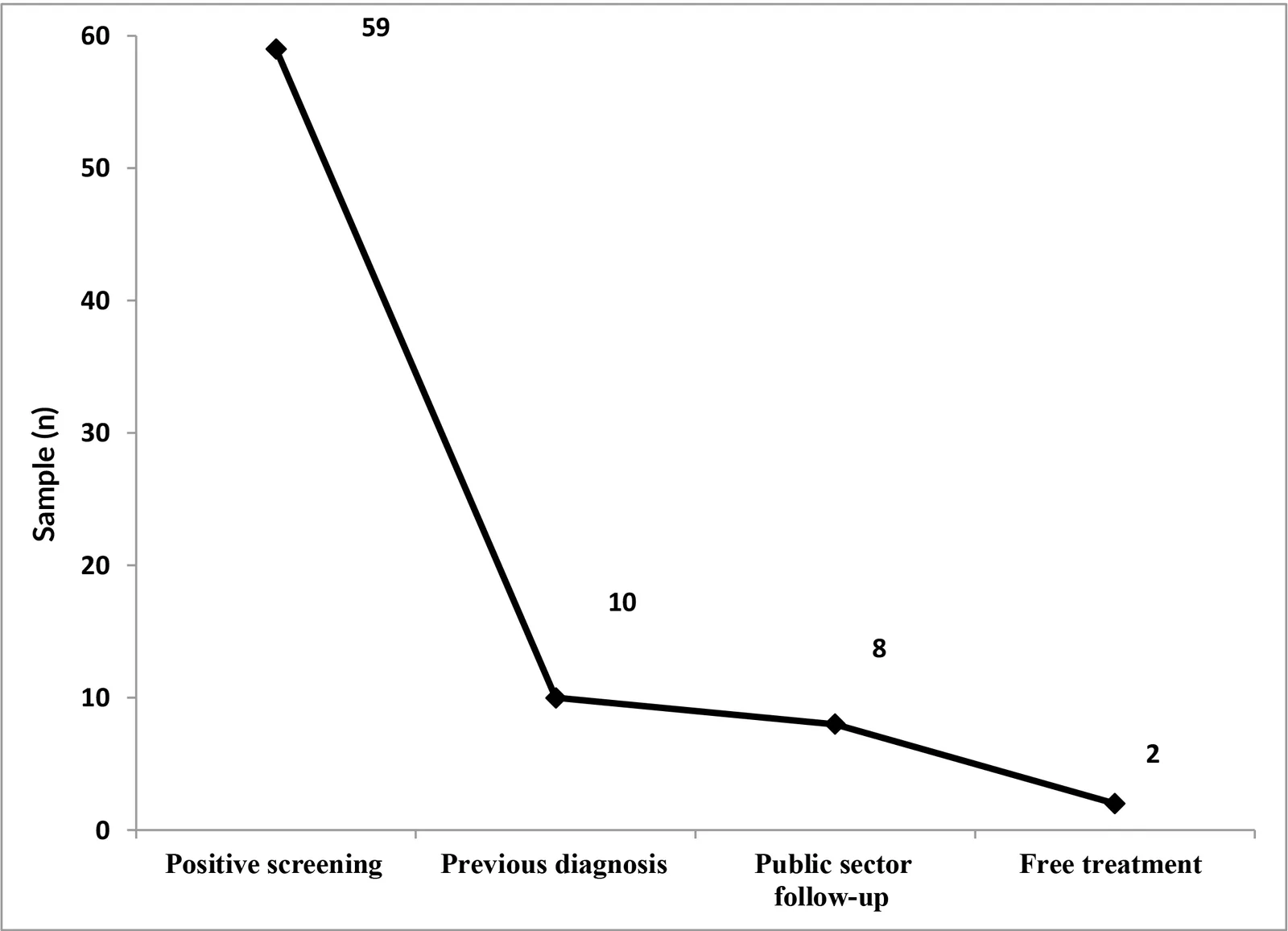
Mental health care cascade of Health Care Network users. Ceará, 2020 (n=277)

Statistically significant association was detected between CMD and: “Consulting a health professional in the last 12 months” (X2[1]=10.578; p-value<0.001); “Previous diagnosis(es) of health problem(s) made by a health professional” (X2[1]= 6.469; p-value 0.008); “follow-up service type” (X2[2]=11.049 p-value 0.004); “method of obtaining medication(s) taken daily” (X2[3]=18.972 p-value<0.001); “overall assessment of the municipality’s public health service” (X2[2]=7.410 p-value 0.025) and “self-rated health” (X2[2]=46.756; p-value<0.001), as described in Table 3.

**Table 3:** Absolute (n) and relative (%) frequencies and association (X2) between presence of common mental disorders and health care provided in the Health Care Network. Ceará, 2020 (n=277)

Variables	Common mental disorders n (%)			X2	p-value
**Sought professional health care in the last 12 months**				10.57	0.001
Yes		47 (27.6)			
**Previous diagnosis of health problem(s) made by health professional**				6.46	0.011
Yes		41 (26.9)			
**Health follow-up type **				11.04	0.004
Does not have follow-up		20 (15.3)			
Exclusively private or complementary			15 (40.5)		
Exclusively public			24 (78)		
**Means of obtaining medication taken every day**				18.97	<0.001
Does not take medication			21 (13)		
Buys medication			20 (32.8)		
Both free and bought			15 (39.5)		
Free			3 (17.6)		
**Overall assessment of the municipal public health service **				7.41	0.025
Poor/very poor			11 (42.5)		
Regular			22 (19.3)		
Good/very good			26 (19.3)		
**Self-rated health**				46.75	<0.001
Poor/very poor			12 (63.2)		
Regular			27 (39.7)		
Good/very good			20 (10.5)		

CMD prevalence was: 2.46 times higher (95%CI 1.37; 4.42; p-value 0.001) in service users who consulted a health professional in the last 12 months; 1.87 times higher (95%CI 1.13; 3.09; p-value 0.011) in people with previous diagnosis(es) of health problem(s) made by a health professional; and 1.84 times higher (95%CI 1.08; 3.11; p-value 0.004) in people who were receiving care in a private service either on an exclusive basis or on a complementary basis.

Presence of CMDs was 2.19 times higher (95%CI 1.24; 3.87; p-value 0.025) among service users who rated the municipality’s public health service as “poor/very poor”. CMD prevalence was higher among individuals reporting self-rated health reported as “poor/very poor”, 6.00 times higher (95%CI 3.50; 10.27; p-value<0.001), and as “regular”, 3.77 times higher (95%CI 2.27; 6.26; p-value<0.001) (Table 4).

**Table 4:** Prevalence ratios (PR) and 95% confidence intervals (95%CI) of presence of common mental disorders in users of services provided by the Health Care Network. Ceará, 2020 (n=277)

Variables	PR (95%CI)	p-value
**Sought professional health care in the last 12 months **		0.001
Yes	2.46 (1.37; 4.42)	
No	1	
**Previous diagnosis of health problem(s) made by health professional**		0.011
Yes	1.87 (1.13; 3.09)	
No	1	
**Health follow-up type**		0.004
Does not have follow-up	0.69 (0.40; 1.18)	
Exclusively private or complementary	1.84 (1.08; 3.11)	
Exclusively public	1	
**Means of obtaining medication taken every day**		<0.001
Does not take medication	0.73 (0.24; 2.22)	
Buys medication	1.85 (0.62; 5.51)	
Both free and bought	2.23 (0.74; 6.71)	
Free	1	
**Overall assessment of ** **the municipal public health service **		0.025
Poor/very poor	2.19 (1.24; 3.87)	
Regular	1.00 (0.60; 1.66)	
Good/very good	1	
**Self-rated health **		<0.001
Poor/very poor	6.00 (3.50; 10.27)	
Regular	3.77 (2.27; 6.26)	
Good/very good	1	

71.5% of participants cited their health center as their most frequently used health service. 38.6% of individuals reported lack of health professional(s) at the health centers. 3.2% of respondents mentioned the Psychosocial Care Center, although none of the study participants reported diagnosis of severe and persistent mental disorder. 

## Discussion

This study provided evidence of a cascade of mental health care for service users in rural municipalities and revealed the reality of patients with health demands, evidenced by the prevalence of CMDs associated with both consulting health professionals in the last 12 months and also with the presence of previous diagnosis(es) of health problem(s) made by a health professional.

Weaknesses were identified in the care provided by the Health Care Network, such as follow-up in public services and obtaining free medication, which is reflected in the users’ assessment of services and in their self-rated health.

Despite the potential of the data presented for guiding changes in mental health care paradigms within the Health Care Network in rural areas, this study has inherent limitations due to its cross-sectional design, using a convenience sample, and being conducted in two specific municipalities in a single state of Brazil, which suggests the need for critical assessment of the results due to the diversity of rural realities.

Underdiagnosis of mental health problems is estimated to exist due to factors such as weaknesses in healthcare or service user difficulty in adhering to longitudinal follow-up. The 2019 National Health Survey (*Pesquisa Nacional de Saúde*, PNS), conducted in a variety of Brazilian municipalities, foud that 73% of individuals questioned about their reasons for seeing a doctor, despite diagnosis of depression, reported that they no longer felt “depressed”, which demonstrates a merely curative-assistential approach. Other reasons reported were: lack of motivation (7%), a very long waiting time at the service (5%), and financial difficulties (2%) (10).

Gaps persist in health care systems, especially in more vulnerable territories, despite advances in recognizing the importance of mental health as an integral part of public health. These difficulties culminate in losses in the cascade of care from diagnosis to treatment. Difficulty in guaranteeing access to and continuity of care, particularly in low- and middle-income countries, can lead to worrying consequences, such as progression to severe symptoms, risk of suicide, or negative repercussions for individuals’ functionality (7,8,16).

The relationship between diagnosed general health problems and CMDs can demonstrate comorbidities between physical illnesses and mental disorders in groups of individuals that are associated with a decline in people’s quality of life, especially among the active working population, such as the participants in this study. In rural populations in China, physical-mental multimorbidity has shown substantial negative effects on the health and economy of individuals, health systems and societies, contributing to higher probability of disability, early retirement, increased sick leave days and lower probability of social participation (17).

The type of service where follow-up takes place is an important ally in individuals’ adherence to treatment, and numerous barriers to access can be seen in rural areas. In the United States, it was identified that 15 older adults living in rural areas reported multiple barriers to services that hindered care, such as limited knowledge of existing services and where to find them, difficulties in obtaining referrals, and unsatisfactory relationships with mental health service professionals, although the participants had shown awareness of the importance of mental health care (18). 

This study demonstrates that service users with CMDs have not been sufficiently assisted in the public network, which leads them to seek supplementary services. The 2019 PNS found the following distribution, in order of prevalence, of types of follow-up services for Brazilians: private clinics (42%); Primary Health Care centers (33%); public hospital/outpatient clinic (9%); Psychosocial Care Center (5%); specialty center, public polyclinic or medical assistance unit (4%); Urgent Care centers; public hospital emergency room (3%); other type of service (2%); private urgent care center or private hospital emergency room (1%) (8,10).

The care of these individuals should be guaranteed in Primary Health Care (PHC), especially through the Family Health Strategy. Primary Health Care centers stood out in this research as the type of health service most used by participants. This level of care has the attributes of longitudinality of care, comprehensiveness of care, family approach, and community orientation to satisfactorily conduct promotion, prevention, treatment and rehabilitation of physical and mental comorbidities of service users registered in the territory, especially those with greater biopsychosocial vulnerabilities. PHC, through the teamwork of the Family Health Strategy, has the potential to maintain these users linked to health services, even those with severe and persistent mental disorders who need integrated follow-up in PHC and specialized care in a Psychosocial Care Center (19,20).

In our research, many users reported lack of health professionals in Primary Health Care centers located in rural areas. Even after the creation of programs that have had a positive impact on retaining these professionals in more vulnerable areas, especially in small municipalities in the North and Northeast regions of Brazil, such as the *Mais Médicos para o Brasil* Program, in practice, many areas still lack these professionals. Constant changes can be observed in the composition of health teams, which makes it difficult to maintain linkage between health professionals and the community (21).

In this study, despite service users consulting health professionals and having previous diagnoses, mainly with physical condition comorbidies, significant difficulties in accessing public services were observed. Strategies such as mental health matrix support (22,23), strengthening interprofessional work (24) and the effective implementation of Multiprofessional Teams (25,26) are promising ways for addressing these weaknesses.

The fact that a large proportion of service users needed to purchase continuous-use medications was another weakness identified, which is particularly concerning given that it involves a vulnerable population sample. A study conducted in Rio Grande do Sul state, Brazil, revealed inequities associated with pharmacological treatment, demonstrating association between the sample that obtained medications free of charge through the public health system and characteristics of lower education, lower income, Black race/skin color, and living in households registered with the Family Health Strategy; while greater access to medications in the private sector was observed among the wealthier, those with higher education, and those of White race/skin color (27).

Identifying these challenges can contribute to the overall assessment of public health services and, consequently, to individuals’ self-rated health. In the present study, these indicators were given poorer ratings in the group of individuals with CMDs, which corroborated other national studies (10,20). The importance of specific public policies is highlighted, such as the National Policy for Comprehensive Health of Rural, Forest and Water Populations, which proves to be a fundamental tool for overcoming the historical inequities that still mark rural territories (28).

This study highlights the prevalence of CMDs among the rural population, associated with gaps in diagnosis, follow-up and treatment offered in these areas. This reinforces the need to expand the mental health care capacity of the Health Care Network, with emphasis on Primary Health Care. With a view to the future, the development of other population-based studies, specifically on rural realities, with different methodological designs, should be considered.

## Data Availability

The database used in this research is not available, as other analyses involving the same study are still in the publication phase. The database had only been made available to the RESS editors/reviewers for assessment purposes.
