# Supplementary material for: Common mental disorders and care provided by the Health Care Network for the rural population: a cross-sectional study, Ceará, Brazil, 2020
Source: Epidemiol Serv Saude. 2026 Jul 20;35:e20250540. doi: 10.1590/S2237-96222026v35e20250540.en (PMC13686391; doi:10.1590/S2237-96222026v35e20250540.en)
Supplement: Translation

# Translation: Transtornos mentais comuns e cuidados ofertados na Rede de Atenção à Saúde para a população rural: estudo transversal, Ceará, 2020

**DOI:** 10.1590/S2237-96222026v35e20250540.pt

**Authors:** Luis Lopes Sombra, Eugênio de Moura Campos, Ricardo José Soares Pontes, Vanira Matos Pessoa

**Affiliations:** 1 Universidade Federal do Ceará Fortaleza Fortaleza CE Brasil Universidade Federal do Ceará, Fortaleza, CE, Brasil; 2 Fundação Oswaldo Cruz Ceará Eusébio Eusébio CE Brasil Fundação Oswaldo Cruz Ceará, Eusébio, CE, Brasil

**Keywords:** Saúde Mental, Saúde da População Rural, Atenção Primária à Saúde, Necessidades e Demandas de Serviços de Saúde, Estudos Transversais

## Abstract

**Objetivo:**

Analisar associação entre presença de transtornos mentais comuns (TMC) e cuidados ofertados pela Rede de Atenção à Saúde para usuários de territórios rurais do Ceará, Brasil.

**Métodos:**

Estudo transversal com amostra por conveniência dos municípios de Nova Oriente e Icapuí, Ceará, Brasil. Após estudo-piloto, aplicou-se formulário sobre avaliação da Rede de Atenção à Saúde e questionário Self-Reporting Questionnaire para rastreio de TMC, com corte ≥7. Realizou-se cálculo amostral e análise estatística, com a inclusão de teste qui-quadrado, razão de prevalência (RP), intervalo de confiança (IC95%) e geração de p-valor.

**Resultados:**

Participaram do estudo 277 usuários, com prevalência de 21,3% para TMC. Desses, 17% tinham diagnóstico prévio, 13,5% estavam em acompanhamento exclusivo em serviço público e 3,3% recebiam todos os psicofármacos gratuitamente. A presença de TMC mostrou-se associada com procura por profissional de saúde nos últimos 12 meses (RP 2,46; IC95% 1,37; 4,42; p-valor 0,001), diagnóstico(s) prévio(s) de problema(s) de saúde realizado por profissional (RP 1,87; IC95% 1,13; 3,09; p-valor 0,011), local de acompanhamento particular exclusivamente/complementar (RP 1,84; IC95% 1,08; 3,11; p-valor 0,004) e avaliação geral do serviço público de saúde do município como ruim/muito ruim (RP 2,19; IC95% 1,24; 3,87; p-valor 0,025). 71,5% dos participantes citaram o posto de saúde como serviço mais utilizado e 40% informaram falta de profissional(is).

**Conclusão:**

Evidencia-se associação de TMC na população rural com lacunas no diagnóstico, no acompanhamento e no tratamento ofertado, o que reforça a necessidade de ampliar a capacidade da Rede de Atenção à Saúde nos cuidados mentais, sobretudo na Atenção Primária.

**Aspectos éticos**

Esta pesquisa respeitou os princípios éticos, obtendo os seguintes dados de aprovação:

Comitê de ética em pesquisa Escola de Saúde Pública do Ceará

Número do parecer 3.372.478

Data de aprovação 5/6/19

Certificado de apresentação de apreciação ética 07802419.2.0000.5037

Registro do consentimento livre e esclarecido Obtido de todos os participantes da pesquisa.

## Introdução

Nas décadas de 1970 e de 1980, impulsionado pela luta de usuários, familiares e profissionais de saúde pelos direitos humanos, principalmente por meio do Movimento da Luta Antimanicomial, o modelo de atenção em saúde mental no Brasil passou por profundas mudanças, o que resultou na Reforma Psiquiátrica. Essas mudanças, além de superar antigos preconceitos e estigmas relacionados aos pacientes com transtornos mentais, foram fundamentais para um manejo adequado de agravos à saúde mental dos brasileiros. A reforma psiquiátrica brasileira trouxe uma nova realidade para os cuidados em saúde mental e permitiu, dentre outros avanços, que os pacientes com transtornos mentais pudessem ser acompanhados dentro da sociedade (1).

A Constituição da República Federativa do Brasil de 1988 inseriu um significativo número de direitos sociais para atender as necessidades fundamentais da população brasileira. O direito à saúde surgiu como efeito do próprio princípio da dignidade da pessoa humana, que concretiza um dos fundamentos do estado democrático de direito, com a determinação de que todo e qualquer cidadão tem direito a acessar os serviços de saúde, independente de gênero, classe, raça/cor da pele, idade e outras características. Torna-se dever do Estado a promoção de políticas públicas, a fim de possibilitar acesso universal igualitário às ações necessárias para a promoção, a proteção e a recuperação da saúde, com a culminância da Lei nº 8.080, que consolidou a criação do Sistema Único de Saúde (SUS) (2,3).

Essa realidade trouxe um inovador processo de gestão e de cuidado em saúde mental e transformou um sistema voltado basicamente para internamentos em hospitais psiquiátricos (manicômios) para uma nova abordagem, pautada na busca pela reinserção dos pacientes na dinâmica familiar, com acompanhamento na Rede de atenção à Saúde, inclusive a Rede de Atenção Psicossocial, composta pela atenção integrada em diversos equipamentos, como centros de atenção psicossocial, serviços de urgência/emergência, leitos em hospitais e clínicas, residência terapêutica e unidades de atenção primária à saúde (1,4,5).

Neste modelo de atenção, a base de organização e a porta de entrada primordial no SUS é a Atenção Primária à Saúde (APS), a qual contém a Estratégia Saúde da Família, responsável por inserir a família como objeto e sujeito do cuidado vinculado aos cuidados longitudinais dos profissionais de saúde. Dessa forma, a APS deve considerar as especificidades de cada território e ser integrada à Rede de Atenção à Saúde, a fim de desempenhar adequadamente as funções de resolutividade e de coordenação dos cuidados em saúde física e mental (6). 

Um dos grandes desafios a serem enfrentados na atualidade é o adoecimento psíquico, com destaque para os chamados transtornos mentais comuns (TMC). Os TMC compõem-se de sintomas como: insônia, esquecimento, dificuldade de concentração, desânimo, fadiga e sentimentos de inutilidade, os quais causam sofrimento psíquico e ruptura do funcionamento normal do indivíduo, apesar de não fecharem critérios para diagnósticos mentais tradicionais. Esses transtornos comprometem significativamente a vida dos indivíduos acometidos e interferem diretamente em múltiplas dimensões, como a capacidade de trabalhar, de manter vínculos sociais, de cuidar de si mesmo e de tomar decisões. Há projeção de que, em 2030, os transtornos mentais serão o problema de saúde mais incapacitante do ser humano (7). A prevalência dos TMC em populações de diferentes países, apesar de ainda subdiagnosticados, encontra-se entre 29% a 35% (8).

Apesar da comprovada importância da APS para a promoção da saúde mental, a prevenção, o tratamento e a reabilitação dos transtornos mentais, ainda são observadas fragilidades, como: pouca integração entre os serviços, insuficiência no trabalho multidisciplinar, debilidade na atenção integral e dificuldade na resolutividade (9). Essas limitações interferem na atenção à saúde mental da população brasileira, especialmente na população rural, que apresenta piores indicadores de acesso aos serviços de saúde (8). Ao se comparar o acompanhamento dos pacientes com diagnóstico de depressão nos territórios rurais e urbanos, no ano de 2019, observou-se que, nos rurais, 48% das pessoas receberam assistência médica para a doença nos últimos 12 meses, enquanto nos territórios urbanos encontrou-se o percentual de 53% (10).

A forma como os serviços de saúde estão organizados também se configura como determinante social da saúde e pode contribuir com condições de vida e de saúde, principalmente em populações com maiores vulnerabilidades, como as que vivem e sobrevivem em territórios rurais. A saúde rural não pode ser desvinculada do funcionamento da saúde pública e coletiva, sobretudo em países em desenvolvimento como o Brasil, que necessitam realizar um uso racional e eficiente dos recursos disponíveis (11). Como agravante para essas situações de vulnerabilidade, os problemas relacionados à saúde mental ainda não conseguem ser abordados ou solucionados plenamente pelos sistemas nacionais de saúde vigentes (12). A relevância deste estudo se mostra na produção de indicadores de saúde sobre um extrato populacional historicamente estigmatizado e vulnerabilizado.

O objetivo deste estudo foi analisar associação entre a presença de TMC e os cuidados ofertados pela Rede de Atenção à Saúde para usuários de municípios rurais do Ceará, Brasil.

## Métodos

### Delineamento do estudo e cenário

Trata-se de estudo com delineamento transversal e abordagem quantitativa realizado com residentes de Novo Oriente e de Icapuí, localizados no Ceará, Brasil, e classificados pelo Instituto Brasileiro de Geografia e Estatística (13) como rurais adjacentes. Utilizaram-se como critérios: densidade demográfica, acesso aos grandes centros urbanos, estruturação de bens/serviços e localização do município. No ano de 2020, Novo Oriente possuía população de 27.453 habitantes e Icapuí de 18.392 indivíduos, com percentuais de população rural estimada, respectivamente, de 48,1%, e, 68,5%. A Tabela 1 apresenta os componentes da Rede de Atenção à Saúde dos municípios (13,14,15).

**Tabela 1:** Componentes (n) da Rede de Atenção à Saúde. Ceará, 2020

Componentes		Novo Oriente n	Icapuí n
Atenção Primária	Unidades de Atenção Primária à Saúde (unidades rurais)	13 (08)	08 (05)
	Equipes da Estratégia Saúde da Família	11	08
	Agentes comunitários	71	37
	Centros de Apoio à Saúde da Família	01	02
Atenção Secundária	Centros de Especialidades	02	00
	Unidades de Pronto Atendimento	01	01
	Centros de Atenção Psicossocial	01	01
Atenção Terciária	Hospitais Gerais	01	01

### Participantes

Amostragem por conveniência com os seguintes critérios de inclusão: indivíduos com faixa etária entre 18 e 64 anos; economicamente ativos com atividade produtiva – agricultura familiar, pesca artesanal ou ambas as atividades; residentes em território do sertão/campo e do litoral/águas há mais de dois anos; integrantes de família adscrita a uma equipe de saúde da família rural.

Foram excluídos do estudo os trabalhadores rurais que estavam afastados de suas atividades laborais. Devido à pandemia ocasionada pelo Covid-19 em 2020, foi criado um protocolo de biossegurança para coleta de dados em campo, com o intuito de diminuir os riscos de transmissão viral, e acrescentaram-se como critérios de exclusão: indivíduos com sintomas de síndrome gripal; indivíduos com aferição de temperatura corporal ≥37,8°C.

### Variáveis

A variável desfecho “presença de TMC” foi rastreada a partir do questionário Self-Reporting Questionnaire, instrumento criado pela Organização Mundial de Saúde e derivado de pesquisas internacionais. Esse questionário foi traduzido, testado e validado no Brasil e tem o intuito de rastrear indivíduos com transtornos mentais comuns (TMC). Possui sensibilidade de 62,9% a 90%, e especificidade de 44% a 95%. É um questionário composto por 20 perguntas com alternativas de “sim” ou “não”, entre as quais quatro são sobre sintomas físicos e 16 sobre sintomas psicoemocionais. O ponto de corte padronizado na maioria dos estudos para rastreio positivo é de sete respostas afirmativas (16).

Os dados sociodemográficos utilizados foram: sexo; faixa etária; situação conjugal; raça/cor da pele; religião; trabalho; renda mensal familiar. Para elaboração da cascata de cuidados, foram analisadas as variáveis independentes de diagnóstico(s) prévio(s), local(is) de acompanhamento de saúde e medicamento(s) de uso contínuo. A partir desses dados gerais, realizaram-se as especificações relacionadas à saúde mental: diagnóstico de transtorno(s) mental(is); local(is) de acompanhamento de saúde mental; psicofármacos(s) de uso contínuo. As variáveis independentes sobre os cuidados em saúde ofertados na rede de atenção à saúde foram analisadas a partir das seguintes perguntas: “Procurou por profissional de saúde nos últimos 12 meses?”; “Possui diagnóstico(s) de problema(s) de saúde realizado por um profissional?”; “Em qual local realiza acompanhamento da saúde?”; “Como obtém os medicamentos que utiliza diariamente?”; “De forma geral, como você avalia o serviço público de saúde do município?”; “Como você avalia o seu estado de saúde?”. 

### Fontes de dados e mensuração

A amostra foi composta mediante a indicação e a mobilização de informantes-chaves da comunidade, pertencentes aos movimentos populares, com a consideração dos critérios de inclusão e de exclusão da pesquisa. A coleta de dados ocorreu durante os meses de setembro e de outubro de 2020 por meio da utilização de formulário constituído por perguntas sobre dados sociodemográficos e avaliação dos serviços de saúde e da aplicação do instrumento Self-Reporting Questionnaire.

O formulário foi elaborado pelos pesquisadores após oficina de oito horas com representantes de movimentos populares do campo e das águas. Posteriormente, o formulário passou por padronização inicial e foi realizada a sua aplicação por dois pesquisadores com quatro indivíduos, sendo dois homens e duas mulheres. Com base nas demandas vivenciadas pelas pesquisadoras da aplicação inicial, o formulário passou por processos de revisão.

No momento da entrevista, utilizaram-se dispositivos móveis que continham o formulário através do aplicativo Open Data Kit. Os dados inseridos no aplicativo geravam automaticamente uma tabela virtual em plataforma de armazenamento, a qual foi acompanhada virtualmente por pesquisador durante todo o período de coleta. 

### Viés

O estudo apresentou vieses inerentes ao delineamento transversal, bem como limitações relacionadas à sua realização em apenas dois municípios de um único estado brasileiro, o que pode restringir a generalização dos resultados. A utilização de uma amostragem por conveniência também reduz a representatividade estatística dos achados. A pesquisa oferece, mesmo assim, importantes contribuições ao aprofundar a compreensão sobre os cuidados em saúde em contextos rurais, o que permite a identificação de padrões locais e a produção de evidências contextualizadas que podem subsidiar políticas públicas e ações mais sensíveis às realidades de territórios vulnerabilizados.

### Tamanho do estudo

O cálculo amostral foi realizado com parâmetros de nível de confiança de 95%, erro amostral de 5% e proporção estimada da variável desfecho na população de 22,58%, com base em teste-piloto realizado em município rural do mesmo estado com indicadores sociodemográficos semelhantes aos municípios selecionados para este estudo (13,14,15). Tal estudo-piloto ocorreu em Fortim, Ceará, com tamanho da amostra de 31 indivíduos moradores de territórios rurais, em que se encontrou a prevalência de TMC de 22,58% (n=7). A amostra mínima estimada foi de 269 indivíduos.

### Métodos estatísticos

A tabela gerada com os dados coletados foi inserida no programa de estatística Statistical Package for the Social Sciences para realização da análise estatística descritiva, com estimativa de frequências absolutas (n) e relativas (%), e da análise estatística analítica.

A estatística analítica foi realizada com teste de qui-quadrado de Pearson (X^2^) ou teste exato de Fisher para identificação da associação de cada uma das variáveis independentes relacionadas à avaliação dos cuidados de saúde com a variável dependente dicotômica de indivíduos com TMC. Também foi aplicada razão de prevalência (RP) e intervalo de confiança de 95% (IC95%), o que gerou seus p-valores, e adotou-se p-valor<0,05 como significância estatística. 

## Resultados

A amostra total de 277 participantes apresentou o seguinte perfil sociodemográfico: predominantemente do sexo feminino (69,7%), faixa etária entre 51-64 anos (36,1%), média de idade de 43 anos, situação conjugal casado(a)/união estável (79,8%). Sobre raça/cor autodeclarada, 67,5% identificaram-se como pardos, 19,5% como brancos e 10,1% como negros. Entre esses participantes, 64,2% identificaram-se como católicos, 60,7% tinha como principal atividade laboral pesca artesanal e 64,3% com renda mensal familiar de até um salário mínimo (Tabela 2).

**Tabela 2:** Distribuição sociodemográfica absoluta (n) e relativa (%) de usuários da Rede de Atenção à Saúde. Ceará, 2020 (n=277)

Variáveis	n (%)
Sexo	
Masculino	84 (30,3)
Feminino	193 (69,7)
**Faixa etária **(anos)	
18-30	47 (17)
31-40	55 (19,8)
41-50	75 (27,1)
51-64	100 (36,1)
**Situação conjugal**	
Casado(a)/união estável	221 (79,8)
Solteiro(a)	39 (14,1)
Divorciado(a)	13 (4,7)
Viúvo(a)	4 (1,4)
**Raça/cor da pele**	
Parda (Morena)	187 (67,5)
Branca	54 (19,5)
Negra	28 (10,1)
Amarela	7 (2,5)
Indígena	1 (0,4)
Religião	
Católica	178 (64,2)
Evangélica	50 (18,1)
Nenhuma	49 (17,7)
Trabalho	
Agricultura familiar	32 (11,5)
Pesca artesanal	168 (60,7)
Agricultura familiar e Pesca artesanal	77 (27,8)
**Renda mensal familiar **(**salários mínimos**)	
Até 1	151 (64,3)
1-2	39 (16,6)
Acima de 2	45 (19,1)

Identificou-se prevalência de 21,3% de usuários com TMC. Apenas 3,6% relataram ter diagnóstico de algum transtorno mental, dos quais foram apontados transtorno de ansiedade e/ou depressão. Desses, todos relataram que estavam em acompanhamento e 80% (n=8) indicaram o atendimento exclusivamente em serviços públicos. Todos estavam em tratamento contínuo com psicofármacos e 75% (n=6) necessitavam comprar algum dos medicamentos.

Na cascata de cuidados dos pacientes com rastreio positivo para transtornos mentais comuns (TMC), apenas 17% já tinham sido diagnosticados com algum transtorno mental por profissional da saúde, 13,5% (n=8) relataram acompanhamento exclusivo em serviço público de saúde e 3,3% recebiam gratuitamente todos os psicofármacos (Figura 1).

**Figura 1:** Cascata de cuidados em saúde mental de usuários da Rede de Atenção à Saúde. Ceará, 2020 (n=277)

Detectou-se associação estatisticamente significativa de TMC com: “Procura por profissional de saúde nos últimos 12 meses” (X^2^[1]=10,578; p-valor<0,001); “Diagnóstico(s) prévio(s) de problema(s) de saúde realizado por profissional” (X^2^[1]= 6,469; p-valor 0,008); “local de acompanhamento da saúde” (X^2^[2]=11,049 p-valor 0,004); “forma de obtenção do(s) medicamento(s) que utiliza diariamente” (X^2^[3]=18,972 p-valor<0,001); “avaliação geral do serviço público de saúde do município” (X^2^[2]=7,410 p-valor 0,025) e “autopercepção de saúde” (X^2^[2]=46,756; p-valor<0,001). Conforme descrito na Tabela 3.

**Tabela 3:** Frequências absoluta (n) e relativa (%) e associação (X2) entre presença de transtornos mentais comuns nos usuários com os cuidados em saúde ofertados na Rede de Atenção à Saúde. Ceará, 2020 (n=277)

Variáveis	Transtornos mentais comuns n (%)			X2	p-valor
**Procura por profissional de saúde ** **nos últimos 12 meses**				10,57	0,001
Sim		47 (27,6)			
**Diagnóstico(s) prévio(s) de problema(s) de saúde realizado por profissional**				6,46	0,011
Sim		41 (26,9)			
**Local de acompanhamento da saúde**				11,04	0,004
Não faz acompanhamento		20 (15,3)			
Particular exclusivo ou complementar			15 (40,5)		
Público exclusivamente			24 (78)		
**Forma de obtenção do(s) medicamento(s) que utiliza diariamente**				18,97	<0,001
Não utiliza			21 (13)		
Comprado			20 (32,8)		
Gratuito e comprado			15 (39,5)		
Gratuito			3 (17,6)		
**Avaliação geral do serviço público de saúde do município**				7,41	0,025
Ruim/muito ruim			11 (42,5)		
Regular			22 (19,3)		
Bom/ótimo			26 (19,3)		
**Autopercepção de saúde**				46,75	<0,001
Ruim/muito ruim			12 (63,2)		
Regular			27 (39,7)		
Boa/muito boa			20 (10,5)		

A prevalência de TMC foi: 2,46 vezes maior (IC95% 1,37; 4,42; p-valor 0,001) nos usuários que procuraram profissional de saúde nos últimos 12 meses; 1,87 vezes maior (IC95% 1,13; 3,09; p-valor 0,011) nas pessoas com diagnóstico(s) prévio(s) de problema(s) de saúde realizado(s) por profissional; e 1,84 vezes maior (IC95% 1,08; 3,11; p-valor 0,004) nas pessoas que faziam acompanhamento em serviço particular exclusivamente ou complementariamente. 

A presença de TMC foi 2,19 vezes maior (IC95% 1,24; 3,87; p-valor 0,025) nos usuários que avaliaram o serviço público de saúde do município como “ruim/muito ruim”. Houve maior prevalência de TMC nos indivíduos com autopercepção de saúde relatada como “ruim/muito ruim”, 6,00 vezes maior (IC95% 3,50; 10,27; p-valor<0,001), e como “regular”, 3,77 vezes maior (IC95% 2,27; 6,26; p-valor<0,001) (Tabela 4).

**Tabela 4:** Razões de prevalência (RP) e intervalos de confiança de 95% (IC95%) da presença de transtornos mentais comuns em usuários com os cuidados em saúde ofertados na Rede de Atenção à Saúde. Ceará, 2020 (n=277)

Variáveis	RP (IC95%)	p-valor
**Procura por profissional de saúde ** **nos últimos 12 meses**		0,001
Sim	2,46 (1,37; 4,42)	
Não	1	
**Diagnóstico(s) prévio(s) de problema(s) de saúde realizado por profissional**		0,011
Sim	1,87 (1,13; 3,09)	
Não	1	
**Local de acompanhamento da saúde**		0,004
Não faz acompanhamento	0,69 (0,40; 1,18)	
Particular exclusivo ou complementar	1,84 (1,08; 3,11)	
Público exclusivamente	1	
**Forma de obtenção do(s) medicamento(s) que utiliza diariamente**		<0,001
Não utiliza	0,73 (0,24; 2,22)	
Comprado	1,85 (0,62; 5,51)	
Gratuito e comprado	2,23 (0,74; 6,71)	
Gratuito	1	
**Avaliação geral do serviço público de saúde do município**		0,025
Ruim/muito ruim	2,19 (1,24; 3,87)	
Regular	1,00 (0,60; 1,66)	
Bom/ótimo	1	
**Autopercepção de saúde **		<0,001
Ruim/muito ruim	6,00 (3,50; 10,27)	
Regular	3,77 (2,27; 6,26)	
Boa/muito boa	1	

71,5% dos participantes citaram o posto de saúde como serviço de saúde mais utilizado. 38,6% dos indivíduos informaram a falta de profissional(is) nos postos de saúde. 3,2% dos entrevistados citou o Centro de Atenção Psicossocial, apesar de nenhum participante do estudo ter relatado diagnóstico de transtorno mental grave e persistente.

## Discussão

Este estudo evidenciou uma cascata de cuidados em saúde mental de usuários em municípios rurais e revelou a realidade de pacientes com demandas em saúde, evidenciada pela prevalência de transtornos mentais comuns (TMC) associada com a procura por profissionais de saúde nos últimos 12 meses e com a presença de diagnóstico(s) prévio(s) de problema(s) de saúde realizado por profissional. 

Fragilidades foram constatadas nos cuidados ofertados na Rede de Atenção à Saúde, como no acompanhamento em serviços públicos e na obtenção gratuita dos medicamentos, o que se reflete na avaliação dos serviços pelos usuários e na autopercepção de saúde.

Apesar da potencialidade dos dados apresentados para mudanças nos paradigmas de cuidados em saúde mental na Rede de Atenção à Saúde em territórios rurais, a pesquisa apresenta limitações inerentes de um delineamento transversal, com amostra por conveniência, e realizada em dois municípios específicos de um único estado do Brasil, o que sugere uma avaliação crítica devido a diversidades das realidades rurais.

Estima-se que exista um subdiagnóstico dos problemas de saúde mental devido a fatores como fragilidades na assistência em saúde ou dificuldade dos pacientes aderirem ao acompanhamento longitudinal. Na Pesquisa Nacional de Saúde (PNS) de 2019, realizada em diferentes municípios brasileiros, 73% dos indivíduos interrogados sobre os motivos para não irem ao médico, apesar do diagnóstico de depressão, referiram não se sentirem mais “deprimidos”, o que demonstra a procura meramente assistencialista-curativa. Outros motivos relatados: não tinham ânimo (7%), tempo de espera em serviço muito grande (5%) e dificuldades financeiras (2%) (10).

Persistem lacunas nos sistemas de atenção, sobretudo em territórios mais vulnerabilizados, apesar dos avanços no reconhecimento da importância da saúde mental como parte integrante da saúde pública. Essas dificuldades culminam em perdas na cascata de cuidados desde o diagnóstico até o tratamento. A dificuldade em garantir o acesso e a continuidade aos cuidados, principalmente em países de baixa e de média renda, pode culminar em consequências preocupantes, como a evolução para sintomas graves, o risco de suicídio ou repercussões negativas para a funcionalidade dos indivíduos (7,8,16).

A relação de problemas gerais de saúde diagnosticados e os TMC podem demonstrar comorbidades entre doenças físicas e transtornos mentais nos grupos de indivíduos que estão associados à piora na funcionalidade de vida das pessoas, principalmente no público de trabalhadores ativos, como nos participantes deste estudo. Em população rural da China, a multimorbidade físico-mental apresentou efeitos negativos substanciais na saúde e na economia dos indivíduos, dos sistemas de saúde e das sociedades, o que contribui para maior probabilidade de incapacidade, de aposentadoria precoce, de aumento de dias de licença médica e de menor probabilidade de participação social (17).

O local de acompanhamento da saúde configura-se como um importante aliado na adesão ao tratamento dos indivíduos e inúmeras barreiras de acesso são observadas nos territórios rurais. Nos Estados Unidos, foi identificado que 15 idosos que viviam em localidades rurais observavam múltiplas barreiras aos serviços que dificultavam o acompanhamento, como conhecimento limitado dos serviços existentes e onde encontrá-los, dificuldades em obter encaminhamentos e relacionamentos insatisfatórios com profissionais de serviços de saúde mental, embora os participantes tivessem mostrado percepção da importância dos cuidados de saúde mental (18).

O presente estudo demonstra que os usuários com TMC não tem sido suficientemente assistidos na rede pública, o que os leva à necessidade de buscar serviços suplementares. A PNS de 2019 constatou a seguinte distribuição, em ordem de prevalência, dos locais de acompanhamento de saúde dos brasileiros: clínica particular ou privada (42%); Unidade de Atenção Primária à Saúde (33%); hospital/ambulatório público (9%); Centro de Atenção Psicossocial (5%); centro de especialidade, policlínica pública ou posto de assistência médica (4%); Unidade de Pronto Atendimento; emergência de hospital público (3%); outro tipo de lugar (2%); pronto-atendimento particular ou emergência de hospital particular (1%) (8,10).

Esses indivíduos devem ter seus cuidados garantidos na Atenção Primária à Saúde (APS), sobretudo por meio da Estratégia Saúde da Família. As Unidades de Atenção Primária à Saúde ficaram em destaque nesta pesquisa como o local de saúde mais utilizado pelos participantes. Esse nível de atenção possui os atributos de longitudinalidade do cuidado, de integralidade da atenção, de abordagem familiar e de orientação comunitária para conduzir de maneira satisfatória a promoção, a prevenção, o tratamento e a reabilitação das comorbidades físicas e mentais dos usuários adscritos no território, principalmente naqueles com maiores vulnerabilidades biopsicossociais. A APS, por meio do trabalho em equipe da Estratégia Saúde da Família, possui a potencialidade de manter o vínculo desse usuário com os serviços de saúde, mesmo daqueles com transtornos mentais graves e persistentes que precisam de acompanhado integrado da APS e da atenção especializada em Centro de Atenção Psicossocial (19,20).

Em nossa pesquisa, muitos usuários relataram falta de profissionais de saúde nas Unidades de Atenção Primária à Saúde localizadas nos territórios rurais. Mesmo após criação de programas que trouxeram impactos positivos na fixação desses profissionais em áreas de maior vulnerabilidade, sobretudo em municípios de pequeno porte do Norte e do Nordeste, como o Programa Mais Médicos para o Brasil, ainda existem, na prática, muitas áreas com carência desses profissionais. Constantes modificações são observadas na composição das equipes de saúde, o que torna frágil a manutenção do vínculo dos profissionais com a comunidade (21).

Neste estudo, apesar da busca por profissionais de saúde e da presença de diagnósticos prévios, principalmente com comorbidades de condições físicas, verificaram-se dificuldades importantes no acesso aos serviços públicos. Estratégias como o matriciamento em saúde mental (22,23), o fortalecimento do trabalho interprofissional (24) e a implementação efetiva das Equipes Multiprofissionais (25,26) são caminhos promissores para enfrentar tais fragilidades. 

A necessidade de compra de medicamentos de uso contínuo por grande parte dos usuários foi outra fragilidade identificada, que é preocupante principalmente por ser com uma amostra populacional vulnerabilizada. Em estudo realizado no Rio Grande do Sul, Brasil, foram reveladas iniquidades associadas ao tratamento farmacológico, que demonstram associação entre a amostra que obteve medicamentos gratuitamente pelo sistema público de saúde e características de menor escolaridade, de menor renda, de raça/cor da pele preta e de residência em domicílios cadastrados na Estratégia Saúde da Família; enquanto o maior acesso aos medicamentos no setor privado foi observado entre os mais ricos, de maior escolaridade e de raça/cor da pele branca (27).

A identificação desses desafios podem contribuir na avaliação geral dos serviços públicos de saúde e, consequentemente, na autopercepção de saúde nos indivíduos. No presente estudo, esses indicadores tiverem piores conceitos atribuídos no grupo de indivíduos com TMC, o que corroborou outros estudos nacionais (10,20). Destaca-se a importância de políticas públicas específicas, como a Política Nacional de Saúde Integral das Populações do Campo, da Floresta e das Águas, que se mostra um aparato fundamental para a superação das iniquidades históricas que ainda marcam os territórios rurais (28).

Este estudo evidencia a prevalência de TMC na população rural associada a lacunas no diagnóstico, no acompanhamento e no tratamento ofertado nesses territórios, o que reforça a necessidadade de ampliar a capacidade da Rede de Atenção à Saúde nos cuidados mentais, com ênfase na APS. Para futuras perspectivas, considera-se o desenvolvimento de outros estudos populacionais, especificamente sobre a realidade rural, com diferentes delineamentos metodológicos.

## Data Availability

O banco de dados utilizado na pesquisa não está disponível, visto que outras análises do estudo estão em fase de publicação. Ele foi disponibilizado de forma restrita aos editores/revisores da RESS para fins de avaliação.
